# Supplementary material for: Saliva Is a Valid Alternative to Nasopharyngeal Swab in Chemiluminescence-Based Assay for Detection of SARS-CoV-2 Antigen
Source: J Clin Med. 2021 Apr 2;10(7):1471. doi: 10.3390/jcm10071471 (PMC8038133; doi:10.3390/jcm10071471)
Supplement: Supplementary file 1 [file jcm-10-01471-s001.pdf]

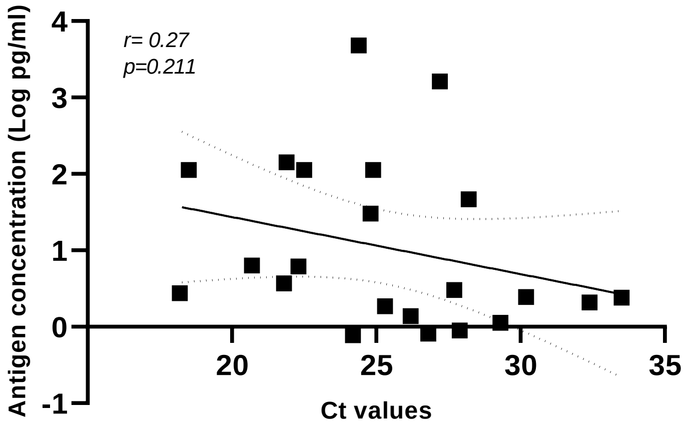

Supplementary Figure S1. Correlation between Ag concentration (Log pg/mL) and Ct values on frozen saliva samples
